# Analysis of homocysteine levels in carriers with *MMACHC* gene variants

**DOI:** 10.1186/s13023-026-04468-2

**Published:** 2026-07-07

**Authors:** Yi Ding, Yuxin Deng, Haijuan Zhi, Xia Zhan, Wenjun Ji, Zizhen Gong, Lili Hao, Lianshu Han

**Affiliations:** Department of Pediatric Endocrinology and Genetic Metabolism, Xinhua Hospital, Shanghai Institute of Pediatric Research, Shanghai Jiao Tong University School of Medicine, 1665 KongJiang Road, Shanghai, 200092 China

**Keywords:** *MMACHC* gene, Carriers, Methylmalonic acidemia, Homocysteine

## Abstract

**Objective:**

Variants in the *MMACHC* gene cause combined methylmalonic acidemia (MMA) and homocystinuria, cobalamin C (cblC) type. This study aimed to determine whether heterozygous *MMACHC* variant carriers exhibit elevated plasma homocysteine (Hcy) levels and to explore the variant-type–specific biochemical effects.

**Methods:**

In this prospective study, 301 *MMACHC* variant carriers and 304 controls were enrolled. Plasma Hcy levels, demographic characteristics, and clinical data were collected. Propensity score matching (PSM, 1:1) was performed to balance age and sex between groups. Generalized linear models were applied to investigate the correlation between *MMACHC* variant carriage and Hcy levels.

**Results:**

After PSM, 173 carrier–control pairs were included. Carriers showed significantly higher Hcy levels (median 12.27 µmol/L, range 5.30–85.90) than controls (9.80 µmol/L, 3.10–53.30; *P* < 0.001). Both Male sex (β = 7.816, 95% CI:5.746–9.887) and *MMACHC* variant carriage (β = 5.741, 95%CI:3.743–7.740) were independently associated with elevated Hcy. Among carriers, missense variants, especially c.80A > G (p.Q27R), were correlated with higher Hcy levels.

**Conclusion:**

Heterozygous *MMACHC* variant carriers had higher plasma Hcy levels than age- and sex-matched controls in this study. Missense variants and male sex tended to be associated with higher Hcy levels. These findings suggest that *MMACHC* variant carriers can present with biochemical abnormalities and associated with alterations in homocysteine metabolism.

**Supplementary Information:**

The online version contains supplementary material available at https://doi.org/10.1186/s13023-026-04468-2.

## Introduction

Combined methylmalonic acidemia (MMA) and homocystinuria, cobalamin C (cblC) type, represents the most common autosomal recessive organic acidemia in China [1]. Globally, the incidence of methylmalonic acidemia (MMA) ranges from 1:20,000 to 1:125,000, while China reports a significantly higher incidence of 1:3,000 to 1:47,000 [2–8]. This disease is caused by variants in the *MMACHC* gene, which plays a role in cobalamin metabolism. *MMACHC* deficiency disrupts the conversion of cobalamin to adenosylcobalamin, leading to the accumulation of methylmalonic acid and methylcitric acid. It also compromises methylcobalamin (MeCbl) synthesis, the cofactor for methionine synthase enzymes, functional impairment in MeCbl compromises the remethylation of Hcy to methionine (Met), resulting in elevated Hcy and reduced Met levels [9–12].

The phenotype of cblC-type MMA is highly heterogeneous with clinical onset ranging from the fetal period to adulthood, often associated with the *MMACHC* variants [13]. For example, severe early-onset cases are frequently associated with variants c.609C > T and c.271dupA, while c.482G > A typically appears in late-onset disease [14, 15]. Diagnosis is mainly based on the results of tandem mass spectrometry (MS/MS), gas chromatography-mass spectrometry, plasma Hcy measurement, and genetic analysis. Standard treatment involves intramuscular vitamin B12 injections combined with oral supplementation of L-carnitine, betaine and folic acid, which achieves partial metabolic improvement, however, Hcy levels improve but rarely normalize [16].

Prognostic studies of cblC deficiency have demonstrated that elevated plasma Hcy levels correlate with reduced developmental quotient [17]. Furthermore, increased Hcy levels are strongly associated with the pathogenesis of multiple disorders, including hydrocephalus, epilepsy, cognitive decline, cardiovascular and cerebrovascular diseases, peripheral vascular disease, cognitive dysfunction, pregnancy complications, neurodegenerative diseases, bone fractures, chronic kidney disease, and adverse pregnancy outcomes [18–21]. Besides *MMACHC* variants, multiple factors influence Hcy levels, including genetic defects in Hcy metabolic enzymes, nutritional deficiencies of vitamin cofactors (folate, B12, B6), comorbidities, environmental exposures, gender differences, and lifestyle factors [22].

Our team previously observed elevated Hcy levels in *MMACHC* variant carriers in clinical practice. In addition, Joyce BJ et al. [23] reported 13 loci associated with plasma total Hcy, including six novel heterozygous variants located in or near *MMACHC*. Based on these findings, the present study investigates the association between *MMACHC* variant carriers and elevated Hcy levels through combined clinical and genotypic analyses.

## Methods

### Subjects

The data collection and patient enrollment were performed between January 2021 and March 2025. A total of 301 carriers with *MMACHC* variants, each of whom had a child diagnosed with cblC-type methylmalonic acidemia, and 304 controls who underwent routine health examinations were enrolled. All participants underwent plasma Hcy level. 257 (85.38%) carriers measured blood values C3, C2, C3/C2 ratio, and 252(83.72%) carriers measured urinary methylmalonic acid and MCA. Information about demography, health-related habits, medical history, past and current diseases and use of medication were collected in carriers. Dietary habits were defined as Regular, Greasy, Light, Salty, Other, or Missing based on the Dietary Guidelines for Chinese Residents (2022).The carriers and control group were propensity score-matched to cases in a 1:1 ratio based on age and sex, using the nearest neighbor method and caliper at 0.01. This process yielded 173 matched carrier-control pairs. Moreover, carriers were divided into normal (Hcy < 15 µmol/L) and abnormal (Hcy ≥ 15 µmol/L) subgroups. Genotype classification of *MMACHC* variants followed the method described in Ding et al. [24]. And Group A comprised the c.80A > G (p.Q27R) variant, Group B comprised c.482G > A (p.R161Q), and Group C comprised c.394C > T (p.R132X), Group D compassed variants predicted to result in truncated or absent MMACHC protein.Each participant provided written informed consent. This study was approved by the Ethics Committee of Xinhua Hospital (Approval No. XHEC-D-2025-169).

### Metabolite detection

Plasma total Hcy levels were measured by fluorescence polarization immunoassay. Blood tandem mass spectrometry (MS/MS; Applied Biosystems, API 4000, California, United States) was employed to measure acylcarnitines (including propionylcarnitine and acetylcarnitine) and amino acids from dried blood spots, with the C3/C2 ratio calculated accordingly [25].Concurrently, organic acids in urine, including methylmalonic acid and methylcitric acid (MCA), were measured using gas chromatography-mass spectrometry (GC/MS; Shimadzu Limited, QP2010, Kyoto, Japan) [1].

### Genetic testing

All *MMACHC* variant sites in carriers were confirmed by Sanger sequencing or next-generation sequencing (NGS). In addition, carriers with plasma Hcy levels ≥ 30 µmol/L (*n* = 28) underwent extended NGS panel testing to explore other potential genetic causes of hyperhomocysteinemia(HHcy)(**Supplementary eTable 1**). Genomic DNA was extracted from peripheral blood using standard protocols. The NGS panel included *CBS*, *MTHFR*, *MTR*, *MTRR*, and *MMACHC*, which are known to be associated with HHcy. All reference sequences were obtained from the NCBI GenBank database. The ClinVar and HGMD databases, along with previously published literature, were consulted to determine whether these variants had been reported. The pathogenicity of novel variants was evaluated according to the standards and guidelines set by the American College of Medical Genetics and Genomics (ACMG).

### Statistical analyses

Statistical analyses were performed using SPSS 25.0 (IBM Corp., Armonk, New York). Non-normally distributed continuous data were described as median (minimum–maximum) or median (interquartile range, IQR), and between-group comparisons were conducted using the Mann-Whitney U test. Categorical data were expressed as counts (percentage), and comparisons between groups were made using the chi-square test or Fisher’s exact test. PSM was used to balance potential prognostic factors, assuming an imbalance in the patient background between the carriers and control groups. The parameters included in the PSM were age and sex, as both are known to influence Hcy levels. The PSM involved 1:1 matching using the nearest neighbor method and caliper at 0.01. A generalized linear model was used for regression analysis. A two-sided *P*-value < 0.05 was considered statistical significance.

## Results

### Characteristics of the participants

This study included a total of 301 carriers of the *MMACHC* gene variants, comprising 138 males (45.85%) and 163 females (54.15%). Based on plasma Hcy levels, 76.08% of all carriers were assigned to the normal group, and 23.92% to the abnormal group. Baseline characteristics according to Hcy status are summarized in Table 1. A significant sex difference was observed in the abnormal group, in which males accounted for 88.89%. There were no statistical differences between the two groups with respect to age, body mass index (BMI), smoking history, alcohol consumption, nutritional habits, dietary, medication or diseases.

**Table 1:** Demographic characteristics of carriers with *MMACHC* Variants

	Total	Normal group (Hcy < 15 µmol/L)	Abnormal group (Hcy ≥ 15 µmol/L)	*P* value
Cases (*n*)	301	229(76.08)	72(23.92)	
sex(male)	138(45.84)	74(32.31)	64(88.89)	< 0.001
Age (years)	35(32,38)	35(32,38)	34.5(31,38)	> 0.05
BMI, n (%)				
Underweight	10(3.32)	10(4.37)	0(0.00)	> 0.05
Normal	131(43.52)	113(49.34)	18(25.00)	
Overweight	88(29.24)	60(26.20)	28(38.89)	
Obese	40(13.29)	20(8.73)	20(27.78)	
Missing	32(10.63)	26(11.35)	6(8.33)	
Baesline of Hcy level (µmol/L) M(range)	11.12 (5.30–85.90)	9.47 (5.30–14.70)	23.16 (15.00-85.90)	< 0.001
Folic acid, B6 intake history (n,%)	10(3.32)	8(3.49)	2(2.78)	> 0.05
Dietary habits				
Normal	169(56.10)	127(55.46)	42(58.33)	> 0.05
Greasy	45(14.90)	32(13.97)	13(18.06)	
Light	29(9.90)	25(27.59)	21(72.41)	
Salty	13(4.30)	7(53.80)	6(46.20)	
Other	13(4.30)	6(46.20)	7(53.80)	
Missing	32(10.60)	16(50.00)	16(50.00)	
Smoking history (n,%)	59(19.60)	32(13.97)	27(37.50)	> 0.05
Fatty liver (n,%)	36(11.96)	21(9.17)	15(20.83)	> 0.05
Alcohol consumption (n,%)	32(10.60)	19(8.30)	13(18.06)	> 0.05
Hypertension (n,%)	21(6.98)	14(6.11)	7(9.72)	> 0.05
Hyperlipidemia (n,%)	13(4.30)	8(3.49)	5(6.94)	> 0.05
Thyroid disorder (n,%)	7(2.32)	0(0.00)	7(100.00)	> 0.05
Diabetes history (n,%)	4(1.30)	4(100.00)	0(0.00)	> 0.05
Renal disease (n,%)	1(0.30)	1(100.00)	0(0.00)	> 0.05
Coronary heart disease (*n*,%)	0(0.00)	0(0.00)	0(0.00)	> 0.05
Gene viriant group				> 0.05
A	26(8.64)	15(6.55)	11(15.28)^a^	
B	18(5.98)	12(5.24)	6(8.33)	
C	8(2.66)	7(3.06)	1(1.39)	
D	210(69.77)	162(70.74)	48(66.67)	
Missing	39(12.96)	33(14.41)	6(8.33)	

a: The proportion is significantly different compared to the normal group (*P* < 0.05)

A total of 301 carriers with *MMACHC* variants and 304 controls were included in this study. Before PSM, there was no significant difference in sex distribution between carriers and controls (*P* = 0.132), but significant differences were observed in age (*P* < 0.001) and age group (*P* < 0.001). The median age was 32 years (28–36.5) in the carrier group and 35 years (32–38.5) in the control group. After 1:1 PSM (caliper = 0.01), 173 matched carrier–control pairs (*n* = 346) were finally included. No significant differences were observed between groups in sex (*P* = 0.911), age (*P* = 0.983), or age group (*P* = 0.852). Baseline characteristics are presented in Supplementary eTable 2. Following PSM, the median plasma Hcy level remained higher in the carrier group [12.27 µmol/L (range 5.30–85.90)] than in the control group [9.80 µmol/L (range 3.10–53.30); *P* < 0.001]. Subgroup analysis by sex revealed that Hcy levels were also higher in both male (*P* < 0.001) and female (*P* < 0.05) carriers than in their respective control groups **(**Table 2**)**.

**Table 2:** Hcy levels in carriers and control group across sex groups

Group	Hcy Level(µmol/L)		
	Total	Male	Female
Carriers	12.27(5.30–85.90)	14.80(8.00-85.90)	9.05(5.30-22.95)
Control	9.80(3.10–53.30)	10.90(6.10–53.30)	7.70(3.10–13.80)
N (Carriers/ Control)	173/173	109/108	64/65
***Z***	-5.818	-6.346	-2.961
***P***	< 0.001	< 0.001	< 0.05

M (minimum-maximum): median (range minimum-maximum)

### Association between Hcy levels and *MMACHC* variants

The generalized linear model analysis was conducted with Hcy levels as the dependent variable, and sex (male), *MMACHC* variant carrier status, and age group as independent variables by using the group after PSM.The model demonstrated a good fit and was statistically significant (Likelihood Ratio Chi-square = 79.317, degrees of freedom = 4, *P* < 0.001). The generalized linear model showed that both male sex (β = 7.816, 95% CI: 5.746–9.887, *P* < 0.001) and *MMACHC* variant carriage (β = 5.741, 95%CI:3.743–7.740, *P* < 0.001) were significantly associated with elevated plasma Hcy levels. In contrast, age group was not significantly correlated with Hcy levels (*P* > 0.05). These findings suggest that both male sex and *MMACHC* variant carriage are independent risk factors for increased Hcy concentration (Table 3).

**Table 3:** Results of generalized linear model analysis

Variable	Regression coefficient B	Standard error	95% CI	Wald χ²	*P*-value
Intercept	5.052	1.2005	[2.699, 7.405]	17.709	< 0.001
Sex (male)	7.816	1.0564	[5.746, 9.887]	54.741	< 0.001
Group (*MMACHC* variant carriage)	5.741	1.0197	[3.743, 7.740]	31.704	< 0.001
Age Group (< 30 years old)	2.381	1.7175	[-0.985, 5.747]	1.922	0.166
Age Group (30–40 years old)	1.259	1.1145	[-0.443, 3.443]	1.276	0.259

### Genetic variant analysis in carriers

Among 262 carriers, 36 distinct *MMACHC* gene variants were identified, including 150 nonsense, 57 missense, 35 deletion, and 20 frameshift variants. The most common variants were c.609G > A (p.W203X), c.80 A > G (p.Q27R), c.658_660delAAG (p.K220del), c.482G > A (p.R161Q), and c.567dupT (p.I190Yfs*13), accounting for 43.9%, 9.9%, 7.6%, 6.9%, and 4.6% of all alleles (Supplementary eTable 3). The median Hcy level among carriers of missense variant was 12.20 µmol/L (range 5.80-78.20µmol/L), the highest among other variant types. Statistical analysis revealed a significant difference in Hcy levels among different variants types (H = 8.303, *P* < 0.05), although pairwise comparisons between individual variant types revealed no significant differences (Table 4**)**. Moreover, The *MMACHC* c.80 A > G (p.Q27R), observed in 26 (45.61%) carriers in missense variant, was significantly more frequent in the abnormal group than in the normal group **(**Table 1**)**, its median Hcy levels was 12.20 µmol/L (range 5.80-78.20 µmol/L).

**Table 4:** Comparison of Hcy levels among carriers with different variants types

Variant Type	Cases (n)	Hcy Level(µmol/L)	Kruskal-Wallis Test	
			H	*P*
Nonsense variant	150	10.01(5.30–84.90)		
Missense variant	57	12.20(5.80–78.20)	8.303	< 0.05
Deletion variant	35	12.20(5.61–40.90)		
Frameshift variant	20	11.80(6.23-57.00)		

M (minimum-maximum): median (range minimum-maximum)

## Discussion

Elevated Hcy levels may be associated with various diseases. This study first found that heterozygous *MMACHC* variant carriers tended to have significantly higher plasma Hcy levels than age- and sex-matched controls, even in the absence of cblC-type MMA. Hcy levels were influenced by both sex and *MMACHC* variant carriage.

Numerous factors can affect plasma Hcy levels. Nutritional status (folate, vitamin B12, and vitamin B6), lifestyle-related factors, renal function, and pathogenic variants in other genes involved in homocysteine metabolism, including *CBS*,* MTHFR*,* MTR*, and *MTRR*, have all been associated with HHcy, whereas smoking, glycosylated hemoglobin and body mass index (BMI) act as indirect risk factors for HHcy [26]. To minimize the confounding effects of these variables, this study used PSM based on age and sex, as these two parameters were verified and available for both groups. Although age differed significantly between the two groups before matching, this difference disappeared after PSM, thereby enhancing the reliability of the findings. After PSM, plasma Hcy levels in the overall carrier group were significantly higher than those in the control group. Correspondingly, the generalized linear model analysis demonstrated that the *MMACHC* variant carriage was positively associated with Hcy levels.

The protein encoded by the MMACHC gene is involved in the metabolism of cobalamin, which functions to transport vitamin B12 from the cytoplasm to mitochondria and convert it into adenosylcobalamin (AdoCbl) and methylcobalamin (MeCbl). Adenosylcobalamin is a cofactor of the methylmalonyl CoA mutase that converts methylmalonyl CoA to succinyl CoA in mitochondria, and methylcobalamin is a cofactor of methionine synthase.

The protein encoded by the *MMACHC* gene is involved in the metabolism of cobalamin, and its function includes converting vitamin B12 into AdoCbl and MeCbl. MeCbl demethylates Hcy to Met. The reason may be related to mild cobalamin metabolic dysfunction caused by reduced *MMACHC* protein expression, while partial loss of *MMACHC* gene function may further disturb the remethylation of Hcy to Met. Supporting this hypothesis, Xin Wang et al. [27] reported in newborn screening that individuals carrying pathogenic genetic variants, although clinically asymptomatic, may exhibit elevated disease-relevant biochemical markers. Meanwhile, Moreno-Garcia et al. [28] reported that heterozygous *Mmachc*^*+/Gt*^ mice showed a 50% reduction of *MMACHC* protein expression and significantly higher levels of plasma Hcy and methylmalonic acid compared with wild-type littermates. These findings indicate that even partial deficiency can impair homocysteine remethylation efficiency. Structurally, the *MMACHC* protein functions in vitamin B₁₂ metabolism by forming multimers or complexes with partner proteins, such as *MMADHC* [29].Variant *MMACHC* monomers may co-assemble with wild-type monomers or other partner proteins, perturbing overall conformation or stability and weakening cofactor catalysis or transfer. These biochemical and molecular findings together suggest that *MMACHC* heterozygosity can lead to subclinical impairment of cobalamin metabolism, manifesting as measurable elevations in plasma Hcy.

A significant sex-based difference in Hcy levels was observed: male carriers had higher levels than both female carriers and male controls, as well as a greater proportion of HHcy cases. The generalized linear model analysis confirmed that male sex independently associated with elevated Hcy levels, consistent with multiple prior studies [30]. Jing Zhao [31] found that among 14,911 Chinese individuals, the upper limit of Hcy levels was below 20 µmol/L in males and 15 µmol/L in females. Potential explanations include higher muscle mass, unhealthy lifestyles (smoking, alcohol use) and lower estrogen levels in men [32, 33].

In the general population, plasma Hcy levels typically increase with age [34], a phenomenon attributed to age-related declines in renal function, reduced vitamin B12 absorption, and alterations in one-carbon metabolism [35]. However, this age-related trend was not evident among carriers in this study. One plausible explanation is that genetic perturbations affecting the cobalamin metabolic pathway may elevate baseline Hcy levels, thereby diminishing the observable effect of aging. Another possible explanation involves compensatory activation of alternative remethylation pathways, such as the betaine–homocysteine methyltransferase (BHMT) pathway, which may partially offset reduced methionine synthase activity. As the BHMT pathway is likely less affected by aging, its compensatory effect could further attenuate the correlation between Hcy levels and age.

Yuan Zeng [36] reported a prevalence of HHcy of 37.2% in the Chinese population based on a meta-analysis. The proportion of HHcy in carriers with *MMACHC* variants was 23.92%. This discrepancy may be attributable to differences in age and population characteristics. Ranran Xu [37] reported that Hcy levels are lowest in individuals aged 30–50 years and increase significantly after the age of 50, with the prevalence of HHcy showing a similar age-related pattern.However, carriers in this study were mainly concentrated in the 30–39 years age range, which overlaps with the age window associated with relatively low Hcy levels. By contrast, the studies included in the meta-analysis covered broader age ranges and included more older individuals, which may have contributed to the higher pooled prevalence of HHcy. Moreover, the 37.2% prevalence was a pooled estimate from 29 heterogeneous studies (I² = 99.8%), with HHcy prevalence varying significantly by age, sex, and region. The carriers was derived from a single center and had a relatively concentrated age distribution. Therefore, the difference between our findings and this highly heterogeneous pooled national estimate may partly reflect differences in study population and design. Notably, HHcy is typically associated with mild elevations in Hcy levels, whereas carriers often exhibit moderate to severe HHcy.

The most common variant was c.609G > A (p.W203X), accounting for 43.9% of all alleles, followed by c.80A > G (p.Q27R) (9.9%), c.658_660delAAG (p.K220del) (7.6%), c.482G > A (p.R161Q) (6.9%), and c.567dupT (p.I190Yfs*13) (4.6%). This distribution is consistent with previous reports in Chinese cblC patients, in which the c.609G > A variant was reported to be associated with early-onset disease, while the c.80A > G variant and c.482G > A variant are more frequently observed in late-onset cases [1, 38].Variant-specific patterns offer additional insight. Carriers of *MMACHC* missense variants, particularly c.80A > G (p.Q27R), tended to exhibit higher Hcy levels than those with other variant types, including nonsense and frameshift variants. It is notable that the c.80A > G (p.Q27R) variant was present in 11 cases (15.28%) among carriers in the abnormal group (Hcy ≥ 15 µmol/L). Among carriers with missense variants, this variant accounted for 26 cases (45.61%). This pattern contrasts with biallelic patient cohorts, where nonsense or frameshift alleles usually confer more severe enzymatic deficiency. In heterozygotes, one normal allele may compensate for a null variant, whereas certain missense substitutions could exert dominant-negative effects that impair function of the wild-type protein through altered folding or dimerization. It is notable that the missense variant c.80A > G (p.Q27R), accounted for 11 cases (15.28%) among carriers with HHcy and 26 cases (45.61%) among carriers with the missense variant. This substitution located within the *MMACHC* N-terminal domain crucial for cobalamin binding, might compromise complex stability and slow cofactor transfer. In addition to the possible dominant-negative effect discussed above, other explanations for the higher Hcy levels observed in missense variants should also be considered. These include the complex genetic backgrounds of carriers, in which modifying genes may increase susceptibility to missense or deletion variants. Alternatively, truncating variants may primarily impair the adenosylcobalamin (AdoCbl) pathway, an effect that could be partially compensated by the remaining normal allele, whereas structural perturbations caused by missense variants may more directly compromise homocysteine remethylation. Although the present data do not prove causality, this variant-type trend justifies further mechanistic work using recombinant expression, structural modeling, and enzyme-activity assays.

This study has several limitations. First, as a cross-sectional analysis, it lacked longitudinal data and repeated Hcy measurements, which may have affected accuracy of the results. Second, limited clinical information was available for the control group, such as vitamin B12 levels, which may have reduced the precision of group matching. Third, *MMACHC* sequencing was not performed in the control group.Fourth, extended family studies involving multiple generations would further strengthen causal inference. Fifth, we did not perform functional experiments to validate the proposed dominant-negative effect of the c.80A > G (p.Q27R) variant.Therefore, the findings require validation in larger, ideally prospective or multicenter cohorts.

## Conclusion

This study demonstrates a significant association between heterozygous *MMACHC* variants and elevated Hcy levels, with many carriers reaching the diagnostic criteria for moderate to severe HHcy. Moreover, male carriers tend to exhibit higher Hcy levels than female carriers. These findings expand the phenotypic spectrum of *MMACHC* variants beyond classical cblC-type MMA, highlighting that carriers who traditionally considered clinically unaffected may exhibit measurable metabolic disturbances. The results emphasize the need to consider genetic background when evaluating unexplained HHcy. Further functional studies are warranted to clarify the molecular mechanisms underlying the incomplete dominance or potential dominant-negative effects of specific *MMACHC* variants.

## Supplementary Information

Below is the link to the electronic supplementary material.

Supplementary Material 1

Supplementary Material 2

Supplementary Material 3

## Data Availability

The data that support the findings of this study are available from the corresponding author upon reasonable request.

## Abbreviations

MMA: Methylmalonic acidemia
cblC: Cobalamin C
Hcy: Homocysteine
C3: Propionylcarnitine
C2: Acetylcarnitine
C3/C2: Propionylcarnitine to acetylcarnitine ratio
MS/MS: Tandem mass spectrometry
GC/MS: Chromatography/mass spectrometry
